# Anti-inflammatory pharmacotherapy in patients with cardiovascular disease

**DOI:** 10.1093/ehjcvp/pvaf058

**Published:** 2025-08-19

**Authors:** Simone Finocchiaro, Placido Maria Mazzone, Nicola Ammirabile, Costanza Bordonaro, Carmelo Cusmano, Luigi Cutore, Giacinto Di Leo, Denise Cristiana Faro, Daniele Giacoppo, Antonio Greco, Antonino Imbesi, Maria Sara Mauro, Carmelo Raffo, Marco Spagnolo, Davide Capodanno

**Affiliations:** Division of Cardiology, Azienda Ospedaliero-Universitaria Policlinico ‘G. Rodolico – San Marco,’ University of Catania, Via Santa Sofia, 78, Catania 95123, Italy; Division of Cardiology, Azienda Ospedaliero-Universitaria Policlinico ‘G. Rodolico – San Marco,’ University of Catania, Via Santa Sofia, 78, Catania 95123, Italy; Division of Cardiology, Azienda Ospedaliero-Universitaria Policlinico ‘G. Rodolico – San Marco,’ University of Catania, Via Santa Sofia, 78, Catania 95123, Italy; Division of Cardiology, Azienda Ospedaliero-Universitaria Policlinico ‘G. Rodolico – San Marco,’ University of Catania, Via Santa Sofia, 78, Catania 95123, Italy; Division of Cardiology, Azienda Ospedaliero-Universitaria Policlinico ‘G. Rodolico – San Marco,’ University of Catania, Via Santa Sofia, 78, Catania 95123, Italy; Division of Cardiology, Azienda Ospedaliero-Universitaria Policlinico ‘G. Rodolico – San Marco,’ University of Catania, Via Santa Sofia, 78, Catania 95123, Italy; Division of Cardiology, Azienda Ospedaliero-Universitaria Policlinico ‘G. Rodolico – San Marco,’ University of Catania, Via Santa Sofia, 78, Catania 95123, Italy; Division of Cardiology, Azienda Ospedaliero-Universitaria Policlinico ‘G. Rodolico – San Marco,’ University of Catania, Via Santa Sofia, 78, Catania 95123, Italy; Division of Cardiology, Azienda Ospedaliero-Universitaria Policlinico ‘G. Rodolico – San Marco,’ University of Catania, Via Santa Sofia, 78, Catania 95123, Italy; Division of Cardiology, Azienda Ospedaliero-Universitaria Policlinico ‘G. Rodolico – San Marco,’ University of Catania, Via Santa Sofia, 78, Catania 95123, Italy; Division of Cardiology, Azienda Ospedaliero-Universitaria Policlinico ‘G. Rodolico – San Marco,’ University of Catania, Via Santa Sofia, 78, Catania 95123, Italy; Division of Cardiology, Azienda Ospedaliero-Universitaria Policlinico ‘G. Rodolico – San Marco,’ University of Catania, Via Santa Sofia, 78, Catania 95123, Italy; Division of Cardiology, Azienda Ospedaliero-Universitaria Policlinico ‘G. Rodolico – San Marco,’ University of Catania, Via Santa Sofia, 78, Catania 95123, Italy; Division of Cardiology, Azienda Ospedaliero-Universitaria Policlinico ‘G. Rodolico – San Marco,’ University of Catania, Via Santa Sofia, 78, Catania 95123, Italy; Division of Cardiology, Azienda Ospedaliero-Universitaria Policlinico ‘G. Rodolico – San Marco,’ University of Catania, Via Santa Sofia, 78, Catania 95123, Italy

**Keywords:** Anti-inflammatory, Pharmacotherapy, Cardiovascular disease

## Abstract

Cardiovascular disease (CVD) remains the leading global cause of morbidity and mortality. In addition to traditional risk factors, inflammation is established as a key mechanism in the initiation, progression, and complications of CVD. Elevated inflammatory biomarkers correlate with disease severity and adverse outcomes, prompting the evaluation of anti-inflammatory therapies in several cardiovascular settings. Colchicine has demonstrated potential in reducing cardiovascular events, though recent trial data have raised concerns regarding its overall benefit and optimal application after myocardial infarction. Alternative agents targeting inflammatory pathways—such as monoclonal antibodies against interleukins (e.g. canakinumab, tocilizumab, ziltivekimab)—have shown biological efficacy but are not yet approved for routine clinical use in CVD. Emerging strategies, including immune-modulatory therapies and RNA-based interventions, seek to achieve selective anti-inflammatory effects with reduced immunosuppressive risk. Future approaches will likely adopt personalized, multi-targeted regimens that integrate inflammation control with lipid-lowering and antithrombotic therapies. As evidence accumulates, inflammation may transition from an adjunctive target to a central focus in CVD management.

## Introduction

Cardiovascular disease (CVD) is the leading cause of morbidity and mortality worldwide, imposing a substantial burden on healthcare systems.^1^ While traditional risk factors—such as hypertension, hypercholesterolaemia, and diabetes—are well recognized and addressed by a broad spectrum of therapeutic strategies, growing evidence highlights the central role of inflammation as a determinant of residual cardiovascular risk, contributing to both the pathogenesis and progression of CVD.^2,3^ Beyond its role in atherogenesis, inflammation is critically involved in plaque destabilization, thrombosis, ventricular remodelling following myocardial infarction (MI), development of heart failure, and progression of certain valvular diseases.^4-7^ The systemic nature of inflammation in CVD is reflected by elevated levels of circulating biomarkers—including high-sensitivity C-reactive protein (CRP), interleukins (ILs), and tumour necrosis factor (TNF)-alpha—which correlate with disease severity and prognosis.^8^

**Figure 1:**
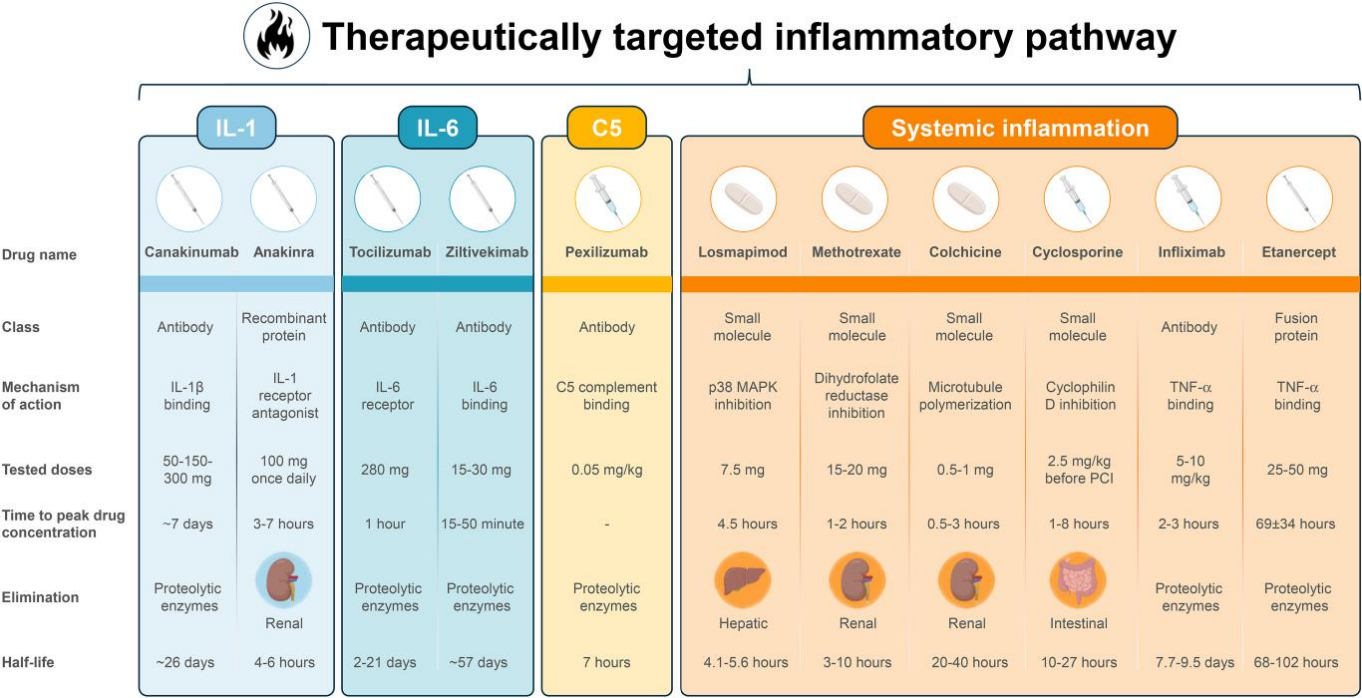
Pharmacokinetics and pharmacodynamics of anti-inflammatory drugs in cardiovascular disease. This figure illustrates the key pharmacokinetic and pharmacodynamic characteristics of various anti-inflammatory drugs used or intended for cardiovascular disease management. IL, interleukin; MAPK, mitogen-activated protein kinase; PCI, percutaneous coronary intervention; TNF, tumour necrosis factor. Pictograms created with BioRender.

The growing recognition of inflammation as a major driver of CVD has spurred increasing interest in therapeutic strategies aimed at modulating immune responses (*Figure 1*). Among the anti-inflammatory agents investigated for cardiovascular applications, colchicine has received particular attention due to its capacity to attenuate inflammation through the inhibition of neutrophil function.^9^ Beyond colchicine, several agents originally developed for immune-mediated disorders have emerged as potential candidates in the CVD domain. Monoclonal antibodies targeting IL ligands or receptors, such as canakinumab (anti-IL-1β), tocilizumab (anti-IL-6 receptor), and ziltivekimab (anti-IL-6), have demonstrated the ability to suppress systemic inflammation, with canakinumab also showing a significant reduction in major adverse cardiovascular events (MACEs) in a large randomized trial.^10-12^ Another agent of special interest is anakinra, a recombinant human IL-1 receptor antagonist that inhibits both IL-1α and IL-1β signalling, thereby blocking upstream inflammatory pathways.^12^

Despite the increasing number of potential anti-inflammatory therapies, several challenges hinder their full implementation in clinical practice and contribute to some degree of scepticism among clinicians. These challenges include the heterogeneity of treatment effects, as anti-inflammatory agents do not appear to exert uniform benefits or a clear class effect. Furthermore, it is essential to identify patient subgroups most likely to benefit, as therapeutic efficacy is unlikely to be universal. Any potential benefit must be weighed against the risk of adverse effects, given that inflammatory responses may play a reparative or physiological role in certain contexts. Therefore, establishing the long-term safety of anti-inflammatory agents in cardiovascular applications remains a critical priority. To guide clinicians through the expanding landscape of investigational therapies, this review examines the role of inflammation across the CVD spectrum, summarizes recent clinical evidence, and discusses how anti-inflammatory strategies may shape future cardiology practice.

## Inflammation and coronary artery disease

### Stable coronary artery disease

Modified lipoproteins, including oxidized low-density lipoproteins, initiate innate immune responses by activating endothelial cells and promoting monocyte recruitment into the intima, where they differentiate into macrophages and foam cells.^13^ This localized inflammation drives coronary artery disease (CAD) progression and destabilization over time. Despite optimal management with lipid-lowering, antithrombotic, and anti-ischaemic therapies, patients with stable CAD remain at significant risk for MACE.^14^ Persistent low-grade chronic vascular inflammation contributes to the progression of atherosclerosis and its thrombotic complications.^15,16^ Growing evidence supporting this pathophysiological link has prompted investigation into targeted anti-inflammatory strategies to reduce cardiovascular risk in patients with stable CAD. Several pharmacological approaches have been assessed, including colchicine, methotrexate, IL-1β inhibitors, and IL-6 inhibitors (*Table 1*).^12,17-20^

**Table 1:** Key randomized controlled trials of anti-inflammatory therapies in patients with stable coronary artery disease

Trial	Study design	Population	Intervention	Active comparator	Primary endpoint(s)	Result
*Colchicine*						
LoDoCo,^18^ 2013	Randomized, observer-blinded	532 Stable CAD	Colchicine 0.5 mg/day	Standard care (no Colchicine)	ACS, fatal or non-fatal out-of-hospital cardiac arrest, or non-cardioembolic ischaemic stroke	HR, 0.33; (0.18–0.59; *P* = 0.001)
LoDoCo2,^19^ 2022	Randomized, double-blinded, placebo-controlled	5522 Stable CAD	Colchicine 0.5 mg/day	Placebo	Cardiovascular death, MI, ischaemic stroke, or ischaemia-driven coronary revascularization	HR, 0.69; (0.57–0.83; *P* = 0001)
*Canakinumab*						
CANTOS,^17^ 2018	Randomized, double-blinded, placebo-controlled	10 061 Stable CAD and hs-CRP ≥ 2 mg/L	Canakinumab 50/150/300 mg every 3 months	Placebo	Non-fatal MI, non-fatal stroke, or cardiovascular death	50-mg group: HR, 0.93; (0.80–1.07; *P* = 0.30) 150-mg group: HR, 0.85; (0.74–0.98; *P* = 0.021) 300-mg group: HR, 0.86; (0.75–0.99; *P* = 0.031)
*Methotrexate*						
CIRT,^20^ 2019	Randomized, double-blinded, placebo-controlled	4786 Prior MI or multi-vessel CAD with DMT2 or metabolic syndrome	Methotrexate 15–20 mg/week	Placebo	Non-fatal MI, non-fatal stroke, or cardiovascular death	HR, 0.96; (0.79–1.17; *P* = 0.001)
*Ziltivekimab*						
ZEUS, NCT05021835	Randomized, quadruple-blinded, placebo-controlled	6200 with CKD, ASCVD, and hs-CRP ≥2 mg/L	Ziltivekimab	Placebo	Cardiovascular death, non-fatal MI, and non-fatal stroke	Expected in 2026

ACS, acute coronary syndrome; CAD, coronary artery disease; ASCVD, atherosclerotic cardiovascular disease; CI, confidence interval; CKD, chronic kidney disease; DMT2, diabetes mellitus type 2; HR, hazard ratio; hs-CRP, high-sensitivity C-reactive protein; MI, myocardial infarction; RCT, randomized controlled trial.

Most studies have focused on colchicine, an anti-inflammatory agent with broad cellular effects, including inhibition of tubulin polymerization and modulation of leukocyte activity.^21^ The LoDoCo study was the first trial to demonstrate the benefit of low-dose colchicine in patients with angiographically confirmed CAD and clinical stability for at least 6 months on optimal medical therapy.^18^ Approximately 11% of patients discontinued treatment early due to gastrointestinal intolerance, a predefined consideration in the protocol and a relevant methodological aspect of the trial. In fact, the protocol envisaged the replacement of patients who had discontinued colchicine due to gastrointestinal side effects within the first month, a decision aimed at maintaining enough patients in the treatment arm. Although these patients were followed for the entire duration of the study and remained in the intention-to-treat analysis, this procedure could raise concerns about potential bias. In particular, early substitution with abandonment could overestimate the real tolerability of colchicine and influence the overall rates of observed events. Over a 3-year follow-up, colchicine significantly reduced the primary composite efficacy endpoint, largely driven by a three-fold reduction in the risk of acute coronary syndromes (ACS). However, the open-label design, small sample size, and lack of a placebo group highlighted the need for larger, controlled trials to confirm these findings.

A few years later, these limitations were overcome by the LoDoCo2 trial, which provided further evidence for the benefit of colchicine.^19^ Among patients with stable CAD for at least 6 months, those with moderate-to-severe renal impairment, severe heart failure, or severe valvular heart disease were excluded. During the initial open-label run-in phase, approximately 15% of patients discontinued treatment due to gastrointestinal side effects. At a median follow-up of 29 months, colchicine reduced the risk of the primary composite endpoint by 31%, primarily driven by a lower incidence of spontaneous MI and ischaemia-driven revascularization. There was no association between colchicine and serious infection or malignancy, and the results were independent of high-sensitivity CRP levels. While the LoDoCo2 study focused on stable CAD patients, it is notable that 85% had a history of ACS. However, a subgroup analysis demonstrated that the benefit of colchicine was consistent regardless of ACS history or timing.^22^

A recent meta-analysis including over 12 000 patients with stable CAD found that colchicine at doses ≤0.5 mg significantly reduced the risk of MI, coronary revascularization, stroke, and cardiovascular hospitalizations compared with placebo, with no difference in cardiovascular or all-cause mortality or gastrointestinal events.^23^ These findings, adding to the results of the LoDoCo2 trials, supported a Class IIa recommendation in the European guidelines for low-dose colchicine (0.5 mg daily) to reduce the risk of MI, stroke, and revascularization in patients with stable CAD.^24^

Different doses of canakinumab were evaluated in stable CAD patients with a prior MI and persistent inflammation (high-sensitivity CRP ≥2 mg/L) in the CANTOS trial.^25,26^ This study is considered a landmark, as it was the first to provide direct evidence supporting the inflammatory hypothesis in atherosclerosis. The 150 mg dose of canakinumab resulted in a 15% reduction in MACE compared with placebo, despite no effect on low-density lipoprotein cholesterol levels, thereby confirming an independent contribution of inflammation to cardiovascular risk. Nevertheless, canakinumab has not been approved for secondary cardiovascular prevention after an MI, likely due to its high cost and the increased incidence of sepsis and fatal infections observed in the trial.^25,26^

In contrast to canakinumab, the folate antagonist methotrexate failed to show cardiovascular benefit in patients with stable CAD, casting doubt on a class effect among anti-inflammatory therapies. Differences in trial design, populations, and treatment timing may partly account for these discrepancies. The CIRT trial tested low-dose methotrexate in patients with CAD and either diabetes or metabolic syndrome.^20^ Methotrexate did not reduce MACE compared with placebo and had no effect on inflammatory biomarkers, suggesting that it does not adequately target the relevant inflammatory pathways in atherosclerosis. The trial was stopped early for futility after a median follow-up of 2.3 years. Notably, participants had lower residual inflammatory risk than in CANTOS, with a median baseline high-sensitivity CRP of 1.5 mg/L (vs. 4.2 mg/L), which may have contributed to the negative findings.

The IL-6 inhibitor ziltivekimab has been evaluated in the phase 2 RESCUE trial, which enrolled patients with chronic kidney disease (CKD) and persistent inflammation (high-sensitivity CRP ≥2 mg/L), a population at high atherosclerotic risk but without confirmed CAD.^12^ Ziltivekimab induced a dose-dependent reduction in high-sensitivity CRP and other inflammation- and thrombosis-related biomarkers, with no significant safety concerns. These results support the hypothesis that IL-6 inhibition may provide a more selective anti-inflammatory strategy than upstream IL-1β blockade by targeting a central mediator of atherosclerosis-related inflammation. Although RESCUE was not powered for clinical endpoints, it provided the rationale for the ongoing phase 3 ZEUS trial (NCT05021835), which is evaluating ziltivekimab in patients with CKD, high-sensitivity CRP ≥2 mg/L, and established atherosclerotic CVD, including stable CAD.

In summary, in patients with stable CAD, residual inflammatory risk persists despite optimal conventional therapies and remains associated with adverse outcomes. Low-dose colchicine has shown a reduction in cardiovascular events in this setting, supporting the hypothesis that targeting vascular inflammation during the chronic phase may be an appropriate use of this drug. In contrast, other anti-inflammatory agents, such as methotrexate, have not demonstrated clinical benefit, likely due to insufficient modulation of key inflammatory pathways implicated in atherosclerosis. Canakinumab reduced events in high-risk patients but was limited by an increased risk of infections and poor cost-effectiveness, underscoring the difficulty of implementing biologic therapies in daily clinical care. Current research in stable CAD is now shifting towards more selective anti-inflammatory strategies, such as IL-6 inhibition with agents like ziltivekimab.

### Acute coronary syndromes

Plaque rupture is a critical event, exposing the necrotic core and causing mechanical disruption that triggers acute inflammation via NOD-, LRR-, and pyrin domain-containing protein 3 (NLRP3) inflammasome activation and subsequent release of IL-1β and IL-18.^27,28^ These cytokines amplify macrophage activation and T-helper 1 polarization, sustaining the inflammatory cascade.^29^ The resulting proinflammatory burst facilitates thrombus formation: Activated platelets interact with the injured endothelium, express P-selectin, and release prothrombotic and inflammatory mediators, thereby linking coagulation with innate immunity.^30^

Following ischaemia, the myocardium exhibits a biphasic inflammatory response—an initial phase that worsens tissue injury, followed by a reparative phase involving regulatory T cells and M2 macrophages.^31,32^ IL-6 functions as a systemic mediator, promoting hepatic CRP production and contributing to endothelial dysfunction, adverse remodelling, and increased long-term cardiovascular risk.^33^

The quest for effective anti-inflammatory therapies in ACS has led to the investigation of multiple pharmacological targets (*Table 2*). Early efforts centred on pexelizumab, a humanized monoclonal antibody directed against complement component C5.^40^ In the COMMA trial, patients with MI were randomized to receive placebo or pexelizumab (administered as a bolus or as a bolus followed by infusion).^41^ While there were no significant differences in infarct size or the 90-day composite endpoint of adverse events, a reduction in mortality was observed in the bolus-plus-infusion pexelizumab group compared with placebo; this finding may reflect a chance effect, as the trial was not powered to assess mortality.^41^ The subsequent COMPLY trial, conducted in patients with ST-segment elevation MI (STEMI) undergoing fibrinolysis, yielded similarly neutral results.^42^ Both COMMA and COMPLY enrolled fewer than 1000 patients, raising concerns about insufficient statistical power to detect a true treatment effect. These limitations were addressed in the larger APEX-AMI trial, which included 5745 patients with acute MI and found no significant differences in 30-day mortality or in the composite endpoint of death, cardiogenic shock, or heart failure at 30 and 90 days between pexelizumab and placebo.^34^ Thus, the initial attempt to validate the inflammatory hypothesis in the setting of MI was unsuccessful.

**Table 2:** Key randomized controlled trials of anti-inflammatory therapies in patients with ACS

Trial	Study design	Population	Intervention	Active comparator	Primary endpoint(s)	Result
*Pexelizumab*						
APEX-AMI,^34^ 2007	Randomized, double-blind, placebo-controlled	5745 STEMI undergoing PCI	Pexelizumab 2.0 mg/kg bolus and placebo infusion for 20 h or pexelizumab 2.0 mg/kg bolus and 0.05 mg/kg/h infusion for 20 h	Placebo	All-cause death	HR, 1.04; (0.80–1.35; *P* = 0.78)
*Cyclosporine*						
CIRCUS,^35^ 2015	Randomized, double-blind, placebo-controlled	970 STEMI undergoing PCI	Cyclosporine 2.5 mg/kg	Placebo	All-cause death, worsening of HF during the initial hospitalization, re-hospitalization for HF, and adverse left ventricular remodelling	OR, 1.04; (0.78–1.39; *P* = 0.77)
*Losmapimod*						
LATITUDE-TIMI 60,^36^ 2016	Randomized, open-label, placebo-controlled	3503 STEMI or NSTEMI	Losmapimod 15 mg/day	Placebo	Cardiovascular death, MI, or severe recurrent ischaemia requiring urgent coronary revascularization	HR, 1.16; (0.91–1.47; *P* = 0.24)
*Colchicine*						
COLCOT,^37^ 2019	Randomized, double-blind, placebo-controlled	4745 AMI undergoing revascularization	Colchicine 0.5 mg/day	Placebo	Cardiovascular death, resuscitated cardiac arrest, MI, stroke, or urgent hospitalization for angina leading to coronary revascularization	HR, 0.77; (0.61–0.96; *P* = 0.02)
COPS,^38^ 2020	Randomized, double-blind, placebo-controlled	795 ACS undergoing PCI or medical therapy	Colchicine 1 mg/day for the first month, then 0.5 mg/day for 11 months	Placebo	All-cause death, ACS, ischaemia-driven urgent revascularization, and non-cardioembolic ischaemic stroke	HR, 0.65; (0.38–1.09; *P* = 0.10)
CLEAR SINERGY,^39^ 2024	2-by-2 factorial design, randomized, placebo-controlled	7062 NSTEMI or STEMI undergoing PCI	Colchicine 0.5 mg/day and/or Spironolactone 25 mg/day	Placebo	Death from cardiovascular causes, recurrent MI, stroke, or ischaemia-driven coronary revascularization	HR, 0.99; (0.85–1.16)
TACTIC, NCT06215989	Randomized, double-blind, placebo-controlled	6574 ACS	Colchicine 0.5 mg/day	Placebo	Cardiovascular death, non-fatal ischaemic stroke, non-fatal spontaneous MI, readmission for ACS, and ischaemia-driven unplanned revascularization	Expected in 2028
COL-BE PCI NCT06095765	Randomized, double-blind, placebo-controlled	2770 ACS and CCS post-PCI	Colchicine 0.5 mg/day	Placebo	All-cause death, spontaneous (non-procedural) MI, stroke, coronary revascularization	Expected in 2028
COLCARDIO-ACS, ACTRN12616000400460	Randomized, double-blind, placebo-controlled	3000 ACS with hs-CRP ≥2 mg/L measured 4–52 weeks post-ACS	Colchicine 0.5 mg/day	Placebo	MI, urgent unscheduled revascularization, cardiovascular death, and non-fatal stroke	Expected in 2029
*Ziltivekimab*						
ARTEMIS, NCT06118281	Randomized, double-blind, placebo-controlled	10 000 (estimated) NSTEMI/STEMI	Ziltivekimab 30 mg loading dose, then 15 mg/month	Placebo	Cardiovascular death, non-fatal MI, non-fatal stroke	Expected in 2026

ACS, acute coronary syndromes; HR, hazard ratio; hs-CRP, high-sensitive C-reactive protein; MI, myocardial infarction; NSTEMI, non-ST-segment elevation myocardial infarction; OR, odds ratio; PCI, percutaneous coronary intervention; STEMI, ST-segment elevation myocardial infarction.

More recently, several established and novel anti-inflammatory agents have been investigated in the setting of MI, but most have failed to demonstrate significant clinical benefit. Cyclosporine, an immunosuppressant that inhibits calcineurin and consequently T-cell activation, initially showed potential in reducing infarct size in a preliminary pilot study.^43^ However, subsequent trials such as CIRCUS and CYCLE, which enrolled patients with STEMI treated within 12 and 6 h of symptom onset, respectively, failed to show improvements in cardiovascular outcomes.^35,44^ Similarly, the larger LATITUDE-TIMI 60 trial evaluated losmapimod, a p38 mitogen-activated protein kinase inhibitor, in patients with non-ST-segment elevation MI (NSTEMI) and additional cardiovascular risk factors.^36^ At 12-week follow-up, losmapimod did not reduce the incidence of the primary composite endpoint—cardiovascular death, MI, or severe recurrent ischaemia requiring revascularization—compared with placebo.^36^

In the COLCOT trial, 4745 patients with a recent MI (mean of ∼13 days prior) were randomized to receive low-dose colchicine (0.5 mg daily) or placebo.^37^ After a median follow-up of 22.6 months, colchicine significantly reduced the incidence of the primary composite endpoint, primarily due to a decrease in stroke and urgent revascularization for angina; however, the rates of recurrent MI did not differ significantly between groups.^37^ The COPS trial subsequently enrolled 795 patients during the index hospitalization for ACS, randomizing them to colchicine 0.5 mg twice daily for 1 month, followed by 0.5 mg daily, or placebo.^38^ This small trial indirectly addressed a critical question regarding the optimal timing of anti-inflammatory therapy initiation. In fact, starting too early may theoretically interfere with acute reparative mechanisms, while delayed initiation may miss the window of maximal benefit. In COPS, at 12 months, the primary composite endpoint did not differ significantly between groups, although a higher all-cause mortality was observed in the colchicine arm; this finding is not consistent with other colchicine trials and lacks mechanistic confirmation.^38^ Moreover, at the extended 24-month follow-up, the colchicine group exhibited a significantly lower incidence of the primary composite endpoint, primarily driven by reduced urgent revascularization, while all-cause mortality was not significantly different between groups.^45^ Overall, COLCOT and COPS initially provided encouraging evidence supporting a role for colchicine in patients with ACS.

The mechanisms underlying the clinical benefit of colchicine in ACS remain speculative but align with the established role of inflammation in this context. Mechanistically, however, there is no conclusive evidence that colchicine directly affects infarct size or post-infarction cardiac remodelling. The COVERT-MI trial investigated the acute effects of colchicine in 192 patients with STEMI undergoing cardiac magnetic resonance imaging.^46^ At 5 days and again at 3 months, no significant differences were observed between colchicine and placebo in terms of infarct size or left ventricular remodelling.^46^ Conversely, trials employing intracoronary imaging have provided partially supportive evidence. The COLOCT trial enrolled 128 patients with ACS and non-culprit vulnerable plaques identified by optical coherence tomography. At 12 months, colchicine was associated with significantly increased minimal fibrous cap thickness, reduced average lipid arc, and decreased macrophage infiltration compared with placebo.^47^ These findings may suggest a potential stabilizing effect on coronary plaques. However, the COCOMO-ACS trial, which included 64 patients with NSTEMI and non-culprit vulnerable plaques, failed to demonstrate significant differences in fibrous cap thickness or lipid arc at a median follow-up of 17.8 months.^48^ These heterogeneous and partially conflicting results indicate that the mechanistic effects of colchicine remain incompletely understood and warrant further investigation.

The current European guidelines on ACS, largely based on the results of the COLCOT trial, indicate that 0.5 mg once daily of colchicine may be considered, particularly if other risk factors are insufficiently controlled or if recurrent cardiovascular events occur despite optimal medical therapy (Class IIb).^49^ However, these guidelines, published in 2023, were developed before the results of the CLEAR trial became available. In this large-scale trial, 7062 patients with MI, predominantly with STEMI (95.1%), were randomized to low-dose colchicine 0.5 mg daily or placebo.^39^ Colchicine and placebo were administered early after MI (median time from symptom onset to randomization 26.8 h). At a median follow-up of 3 years, the primary composite efficacy endpoint was not significantly different between treatment groups, despite a significant reduction in CRP levels in patients assigned to colchicine.^39^ Non-cardiovascular death was significantly lower in the colchicine group compared with the placebo group, though the reason for this difference remains unclear.^39^ All the other individual endpoints, including cardiovascular death, MI, and stroke, were not significantly different between treatment groups.^39^

It is important to consider that the CLEAR trial was conducted during the COVID-19 pandemic, a period characterized by significant disruption to healthcare systems. In a subgroup analysis stratified by pandemic phase, colchicine demonstrated a 22% relative risk reduction in the pre-COVID-19 cohort, consistent with previous trials such as COLCOT, while this benefit was not observed during the pandemic phase. Although the interaction test did not reach formal statistical significance (*P* = 0.098), these findings suggest that the pandemic context may have influenced the overall neutral outcome. Additionally, the drug appeared to show better results in patients receiving a higher dose. Initially, patients weighing >70 kg were prescribed colchicine at 0.5 mg twice daily, but a protocol amendment transitioned all patients to 0.5 mg daily after 90 days due to high discontinuation rates. Notably, a subgroup analysis revealed a trend towards benefit with the higher dose, raising concerns regarding the adequacy of the colchicine dose used in the trial. Given these uncertainties, further research is needed to clarify the role of colchicine in ACS. In this regard, the results of the ongoing large-scale TACTIC (NCT06215989), COL-BE PCI (NCT06095765), and COLCARDIO-ACS (ACTRN12616000400460) trials are awaited.

Beyond colchicine, other selective medications targeting the inflammatory response in patients with recent ACS could open new therapeutic avenues (*Table 1*). Initially approved for rheumatoid arthritis, the potential role of the IL-1α/β inhibitor anakinra has been explored in pilot studies^50,51^ and in the VCU-ART 3 trial, with reductions in CRP and improved cardiac function.^52^ However, the MRC-ILA trial reported increased MACE in NSTEMI patients,^53^ possibly suggesting that the efficacy of anakinra may vary based on the inflammatory burden. In a recent study, the novel IL-1 inhibitor goflikicept significantly reduced high-sensitivity CRP values at 14 days compared with placebo in patients with STEMI.^54^ It is still premature to draw definitive conclusions about this molecule, considering that other anti-inflammatory drugs capable of significantly reducing high-sensitivity CRP have not demonstrated corresponding clinical benefits.

An interesting research question in ACS concerns which part of the inflammatory cascade is most beneficial to antagonize. Acting too upstream may lead to less selective effects and potentially dangerous off-target consequences due to interference with the immune system. Unlike IL-1 inhibition, which affects a wide range of immune cells and may impair immune responses to infections and other stimuli, IL-6 inhibition is more selective. Tocilizumab reduced inflammatory markers and showed signals of improved myocardial salvage in patients with NSTEMI and STEMI,^55^ although no significant difference in infarct size was observed.^11^

The ongoing phase 3 ARTEMIS trial (NCT06118281) will provide insights into the clinical effects of the IL-6 inhibitor ziltivekimab compared with placebo in approximately 10 000 patients with recent acute MI. Additionally, the proof-of-concept phase 2b GOLDILOX-TIMI 69 trial (NCT04610892) will evaluate the efficacy, safety, pharmacokinetics, and immunogenicity of golocdacimab, a fully human monoclonal antibody targeting LOX-1, a receptor primarily expressed in endothelial cells that plays a crucial role in the uptake of oxidized low-density lipoproteins and contributes to atherosclerosis and CVD. These two trials will add clues on whether targeting downstream components of the inflammatory cascade, rather than upstream, is a preferable therapeutic strategy, although it is important to note that their comparison will be against placebo, and these trials will not provide a direct comparison between different antagonists of the inflammatory cascade.

In summary, multiple trials of anti-inflammatory strategies in ACS patients (e.g. pexelizumab, losmapimod) have failed to consistently demonstrate significant clinical benefits. However, most of these studies were underpowered for clinical outcomes; thus, while a lack of effect cannot be excluded, it remains inconclusive. On the other hand, evidence regarding colchicine is mixed. The two largest trials yield opposite results, with the most recent one showing no effect. Despite reducing CRP levels, colchicine has not yet shown a significant effect on cardiovascular mortality, and its routine use is not recommended in ACS. However, selective use is permitted by current European guidelines, which will likely undergo revision to become even more restrictive following the CLEAR trial, at least in patients with STEMI. Several additional trials of colchicine are ongoing, and when combined with the existing evidence, they will help clarify the role of this drug in secondary prevention. ARTEMIS, the large trial of ziltivekimab, is likely the most eagerly awaited piece of information, as downstream inhibition of IL-6 could offer the right balance between safety and efficacy in ACS.

### Microvascular dysfunction

The inflammatory background, in conjunction with traditional cardiovascular risk factors, contributes significantly to the pathophysiology of emerging entities, such as angina with no obstructive coronary artery disease (ANOCA), ischaemia with no obstructive coronary artery disease (INOCA), and myocardial infarction with no obstructive coronary artery disease (MINOCA), which are conditions characterized by myocardial angina, ischaemia, or MI in the absence of obstructive CAD.^56,57^ In MINOCA, inflammation is implicated in multiple pathophysiological mechanisms, including not only coronary microvascular dysfunction but also plaque disruption and epicardial coronary spasm. The degree of microvascular dysfunction in ANOCA, INOCA, and MINOCA correlates with inflammatory markers, such as high-sensitivity CRP and soluble CD40 ligand.^58-60^ Endothelial dysfunction further exacerbates inflammation, promoting immune dysregulation and atherosclerotic progression, with key roles played by IL-1, IL-6, and TNF-α.^61,62^

Despite increasing evidence linking inflammation to ANOCA, INOCA, and MINOCA, anti-inflammatory therapies are not yet established in these settings due to the absence of randomized outcome data. However, pilot studies are beginning to explore this approach in selected patients. The COL-Micro-HF trial (NCT06217120) is assessing colchicine vs. placebo in heart failure and preserved ejection fraction (HFpEF) patients with coronary microvascular dysfunction, using coronary flow reserve as the primary endpoint over 6 months. Similarly, the STRONG trial (NCT06600178) is evaluating the effect of SGLT2 inhibitors on coronary microvascular dysfunction in women with ANOCA, based on their potential to reduce systemic inflammation and improve coronary perfusion.^63^

## Inflammation and heart failure

Inflammation plays a significant role in the pathogenesis and progression of heart failure through a variety of mechanistic pathways, driving myocardial fibrosis, hypertrophy, and dysfunction.^64^ Activation of Toll-like receptor 4 by damage- and pathogen-associated molecular patterns initiates the NLRP3 inflammasome, leading to increased levels of proinflammatory cytokines, such as TNF-α and IL-6.^64^ Endothelial inflammation disrupts nitric oxide bioavailability, stiffens titin, and accelerates fibrosis. The presence of autoantibodies, elevated free light chains, and adipose-derived cytokines further amplifies myocardial injury, while monocytes from the spleen contribute to ongoing inflammation.^64^ Although these findings highlight inflammation as a promising therapeutic target, clinical evidence supporting the efficacy of anti-inflammatory treatments in heart failure remains insufficient (*Table 3*). As such, current European guidelines do not recommend the routine use of anti-inflammatory therapies in this setting.^68^

**Table 3:** Key randomized controlled trials of anti-inflammatory therapies in heart failure

Trial	Study design	Population	Intervention	Active comparator	Primary endpoint(s)	Result
*Etanercept*						
RENEWAL,^65^ 2004	Randomized, double-blinded, placebo-controlled	2048 HFrEF, NYHA 2-4	Etanercept 25 mg/week	Placebo	All-cause death, hospitalization for HF	RR, 1.10 (0.91–1.33; *P* = 0.33)
*Immunomodulation*						
ACCLAIM,^66^ 2006	Randomized, double-blinded, placebo-controlled	2426 HFrEF and HHF, NYHA 2-4	Non-specific immunomodulation on days 1–2–14, and every 28 days	Placebo	All-cause death, cardiovascular hospitalization	HR, 0.92 (0.80–1.05; *P* = 0.22)
*Canakinumab*						
CANTOS (prespecified exploratory analysis),^67^ 2019	Randomized, double-blinded, placebo-controlled	10 054 and 5058 prior MI and baseline hs-CRP and IL-6 available	Canakinumab 50/150/300 mg every 3 months	Placebo	HF-related death, hospitalization for HF	50-mg group: 1.00 (0.78–1.29; 150-mg group: 0.88 (0.68–1.13); 300-mg group: 0.78 (0.60–1.02) *P* for trend = 0.042
*Ziltivekimab*						
HERMES, NCT05636176	Randomized, quadruple-blinded, placebo-controlled	5600 HFpEF/mrEF NYHA 2–4, NT-proBNP ≥300 pg/mL, hs-CRP ≥2 mg/L	Ziltivekimab	Placebo	Cardiovascular death, hospitalization, or urgent visit for HF	Expected in 2027

HF, heart failure; HFmrEF, heart failure with mildly reduced ejection fraction; HFpEF, heart failure with preserved ejection fraction; HFrEF, heart failure with reduced ejection fraction; HR, hazard ratio; hs-CRP, high-sensitivity C-reactive protein; NYHA, New York Heart Association; RR, relative risk.

An exploratory analysis of the CANTOS trial suggested a dose-dependent association between canakinumab therapy and reductions in hospitalization for heart failure and the composite outcome of hospitalization and heart failure-related mortality in patients with prior MI and elevated high-sensitivity CRP levels.^67^ However, CANTOS was not a study focused on heart failure, where the evidence so far has been quite disappointing. Among anti-TNF-α agents, infliximab and etanercept have been evaluated in patients with heart failure and reduced ejection fraction (HFrEF). In a phase 2 trial, infliximab did not improve short-term clinical outcomes despite reductions in inflammatory markers, and, at the highest doses studied, it was associated with increased mortality and heart failure-related hospitalizations.^69^ Etanercept was evaluated in a phase 3 trial, which was prematurely terminated due to a lack of significant clinical benefits, including effects on mortality and hospitalizations.^65^ In phase 2 trials, the IL-1 inhibitor anakinra did not significantly reduce mortality or hospitalization despite its effects on aerobic exercise capacity and systemic inflammation. In HFpEF, clinical benefits were inconsistent and limited to specific subgroups, such as obese patients, while in acute heart failure, improvements in exercise capacity were not accompanied by a significant reduction in MACE.^70-73^

Studies evaluating non-selective anti-inflammatory agents have also demonstrated disappointing results.^66^ In particular, colchicine therapy for 6 months has not shown significant improvements in functional class, mortality, or hospitalization in patients with chronic heart failure.^74^ Similarly, no clinical benefit of colchicine was observed in preventing clinical deterioration in patients with acute heart failure.^75^ Nevertheless, several ongoing clinical trials are currently assessing colchicine in HFpEF (NCT05637398; NCT06130059; NCT06081049; NCT06604611), acutely decompensated HFrEF (NCT06286423), and chronic inflammatory cardiomyopathy (NCT06158698). Small-scale studies assessing methotrexate and thalidomide in heart failure have shown mixed results, with variable effects on functional class, 6-min walking distance, and TNF-α levels.^76-79^

Large-scale outcomes trials of IL-6 inhibition that are currently ongoing include the HERMES trial (NCT05636176), a phase 3 study enrolling approximately 5600 patients with HFpEF or heart failure with mildly reduced ejection fraction and systemic inflammation, evaluating the effect of monthly ziltivekimab injections on clinical outcomes, such as cardiovascular death, hospitalization, and urgent heart failure visits. In parallel, the ATHENA trial (NCT06200207), a phase 3 study of 680 patients with HFpEF and elevated high-sensitivity CRP, is testing the impact of ziltivekimab on quality of life as a primary outcome, alongside key cardiovascular endpoints.

Other small-to-medium-sized studies with surrogate endpoints include REPAIR-CS (NCT06660732), which is testing rilonacept (a dimeric fusion protein inhibiting IL-1β) in cardiac sarcoidosis; CIT (NCT05946772), which is testing cyclosporine in Takotsubo syndrome; STERO-HF (NCT05809011) and CORTAHF (NCT05916586), which are evaluating corticosteroids in acute heart failure; and CYCLONE-LVAD (NCT04596813), which is exploring extracorporeal IL-6 removal in recipients with left ventricular assist devices.

In summary, inflammation contributes to the pathogenesis of heart failure through multiple pathways. Despite a strong mechanistic rationale, clinical trials of anti-inflammatory therapies in heart failure have yielded largely negative or inconclusive results. Agents targeting TNF-α (infliximab, etanercept) and IL-1 (anakinra) failed to demonstrate clinical benefit, with some associated with harm. Non-selective therapies, such as colchicine and methotrexate, have also not been shown to improve key clinical outcomes. Nonetheless, ongoing phase 3 trials, such as HERMES and ATHENA, are ongoing to evaluate IL-6 inhibition with ziltivekimab and may clarify whether more targeted approaches can provide clinical benefit in the heart failure scenario.

## Inflammation and valvular heart disease

Inflammation is increasingly recognized as a key contributor to the pathogenesis of valvular heart disease, particularly valvular stenosis.^80^ While the incidence of rheumatic mitral stenosis has declined, aortic stenosis remains common and carries significant prognostic implications.^81^ Mechanistically, endothelial disruption in aortic stenosis promotes cytokine release, recruitment of inflammatory cells, and activation of valve interstitial cells, which adopt osteogenic phenotypes under proinflammatory stimuli, ultimately leading to leaflet calcification and progressive stenosis.^82^ These insights have raised interest in targeted anti-inflammatory therapies to slow disease progression. However, no pharmacological agents are currently approved for the treatment of aortic stenosis, and the therapeutic potential of immunomodulation remains uncertain.

The causal relationship between inflammation and aortic stenosis has been explored using Mendelian randomization to estimate the genetically proxied effects of tocilizumab, canakinumab, and colchicine, based on data from two large European genome-wide association studies comprising over 500 000 individuals each.^83^ This analysis found that genetically proxied IL-6 inhibition via tocilizumab was associated with a reduced risk of aortic stenosis, while canakinumab showed no significant association. Only one suitable single-nucleotide polymorphism was available to proxy colchicine exposure, limiting interpretability for this agent.^83^ In the Co-STAR trial (NCT04870424), which enrolled patients with severe aortic stenosis undergoing transcatheter aortic valve implantation, low-dose colchicine halved the incidence of significant (≥1) prosthetic leaflet thickening compared with placebo, as assessed by the prespecified 4D-cardiac computed tomography endpoint.^84^ This effect may reflect mitigation of local inflammatory responses induced by mechanical injury to the valve landing zone during deployment, a process thought to contribute to subclinical leaflet pathology.

Despite these findings, no randomized trials have yet evaluated anti-inflammatory agents for native aortic stenosis, a condition for which delaying progression from moderate-to-severe stages would be of clear clinical benefit.^85,86^ Two small trials are currently investigating colchicine in this context. The CHIANTI trial (NCT05162742) is a multicenter, randomized study enrolling patients with moderate aortic stenosis to receive colchicine 0.5 mg daily or placebo, with changes in aortic valve calcium score, ^18^F-sodium fluoride uptake, and peak aortic jet velocity over 24 months as key efficacy endpoints. Similarly, the COPAS trial (NCT05253794), a single-center, double-blind, randomized study, is enrolling patients with mild-to-moderate aortic stenosis to test whether colchicine reduces valvular calcification, with ^18^F-sodium fluoride uptake at 6 months as the primary outcome.

Overall, while strong mechanistic and preliminary clinical evidence supports further investigation of anti-inflammatory therapies in aortic stenosis, ongoing clinical trials are limited to colchicine, despite genetic data suggesting a potential benefit of IL-6 inhibition with agents such as tocilizumab.

## Inflammation, pericarditis and myocarditis

Inflammation is central to the pathogenesis of both pericarditis and myocarditis, and anti-inflammatory therapy remains the cornerstone of their clinical management. While treatment protocols for pericarditis are well established (*Table 4*), the field of inflammatory heart disease is undergoing rapid evolution, driven by novel non-invasive imaging modalities that enable detection and longitudinal monitoring of myocardial inflammation.^96^

**Table 4:** Key randomized controlled trials of anti-inflammatory therapies in patients with pericarditis and myocarditis

Trial	Study design	Population	Intervention	Active comparator	Primary endpoint(s)	Result
*Pericarditis*						
CORE,^87^ 2005	Randomized, open-label, parallel group	84 recurrent pericarditis (first episode)	Colchicine 1–2 mg (loading dose); 0.5–1 mg/day for 6 months	Standard care	Recurrent rate at 18 months	ARR, 26.6%; 24.0% vs. 50.6%; *P* = 0.04; NNT 4.0; (2.5–7.1)
COPE,^88^ 2005	Randomized, open-label, parallel group	120 acute pericarditis	Colchicine, 1–2 mg (loading dose); 0.5–1 mg/day for 3 months	Standard care	Recurrent rate at 18 months	10.7% vs. 32.3%; *P* = 0.004; NNT, 5; (3.1–10)
CORP,^89^ 2011	Randomized, double-blind placebo-controlled	120 recurrent pericarditis (first episode)	Colchicine, 1–2 mg (loading dose); 0.5–1 mg/day for 6 months	Placebo	Recurrent rate at 18 months	ARR, 0.31; (0.13–0.46). RR 0.56; (0.27–0.73; *P* < 0.001) NNT, 3 (2–7)
ICAP,^90^ 2013	Randomized, double-blind, placebo-controlled	240 acute pericarditis	Colchicine 0.5–1 mg/day for 3 months	Placebo	Incessant or recurrent pericarditis	RR, 0.56; (0.30–0.72; *P* < 0.001); NNT, 4
CORP-2,^91^ 2014	Randomized, double-blind placebo-controlled	240 recurrent pericarditis (two or more)	Colchicine 0.5–1 mg/day for 6 months	Placebo	Recurrent pericarditis	RR, 0.49; (0.24–0.65; *P* < 0.009); NNT, 5
COPPS-2,^92^ 2014	Randomized, double-blind, placebo-controlled	360 pericardiotomy	Colchicine 0.5–1 mg/day	Placebo	Occurrence of PPS within 3 months	19.4% vs. 29.4% AD 10.0%; (1.1–18.7%); NNT, 10
Sambola et al.,^93^ 2019	Randomized, open-label	110 first episode of acute idiopathic pericarditis	Colchicine 0.5–1 mg/day for 3 months	Standard care	Recurrent episodes of pericarditis	13.5% vs. 7.8%; *P* = 0.34
RHAPSODY,^94^ 2021	Randomized, double-blind, placebo-controlled	86 acute recurrent pericarditis and elevated CRP levels	Rinolacept 320 mg (loading dose), 160 mg/week	Placebo	Median time to first pericarditis recurrence	Not be estimated vs. 8.6 (4.0–11.7); HR, 0.04; (0.01–0.18; *P* < 0.001)
*Myocarditis*						
ARAMIS,^95^ 2023	Randomized, double-blinded placebo-controlled	120 acute myocarditis	Anakinra 100 mg/day	Placebo	Number of days alive free of any myocarditis complications	30–32 vs. 30–32; *P* = 0.168
MYTHS, NCT05150704	Randomized, single-blinded placebo-controlled	288 acute myocarditis	Methylprednisolone 1 g IV/day for 3 days	Placebo	All-cause death, HTx, LVAD implant, ventricular arrhythmias, first re-hospitalization due to HF or ventricular arrhythmias, or advanced atrioventricular block	Expected in 2028

AD, absolute difference; ARR, absolute risk reduction; CI, confidence interval; CRP, C-reactive protein; HF, heart failure; HR, hazard ratio; HTx, heart transplantation; LVAD, left ventricular assist device; NNT, number needed to treat; OR, odds ratio; PPS, post-pericardiotomy syndrome; RR, risk ratio; RRR, relative risk reduction.

### Pericarditis

Acute and recurrent pericarditis are primarily managed with non-steroidal anti-inflammatory drugs or aspirin, in combination with colchicine at low, weight-adjusted doses (0.5 mg once or twice daily) to improve remission rates and reduce recurrence.^97,98^ In corticosteroid-requiring cases, prednisone may be used as adjunctive therapy but should always be combined with colchicine and non-steroidal anti-inflammatory drugs to minimize the risk of chronicity.^99^ For patients with colchicine-resistant and steroid-dependent disease, second-line immunomodulatory agents, such as intravenous immunoglobulin, anakinra, or azathioprine, can be considered.^100-102^ The RHAPSODY trial demonstrated that rilonacept, an IL-1α/β inhibitor, significantly reduced pericarditis recurrences and enabled rapid tapering of other anti-inflammatory agents.^94^

In summary, the management of pericarditis is guided by established therapeutic algorithms, with non-steroidal anti-inflammatory drugs and colchicine as first-line therapy and newer agents, such as rilonacept, reserved for refractory cases.

### Myocarditis

Myocarditis encompasses a heterogeneous group of disorders with variable etiology, clinical presentation, and outcomes, making treatment more complex and less standardized. Management strategies are largely based on expert consensus, given the paucity of controlled clinical trials.^96,103^ In immune checkpoint inhibitor-associated myocarditis, immediate withdrawal of the offending agent and high-dose intravenous corticosteroids constitute first-line therapy, although mortality remains high. In refractory cases, targeted immunosuppression with agents such as abatacept (CTLA-4 agonist), alemtuzumab (anti-CD52), or antithymocyte globulin (anti-CD3), may be considered.^104-106^ For eosinophilic myocarditis, management includes prompt identification and withdrawal of the causal agent, initiation of corticosteroids, and etiology-specific treatments, e.g. albendazole for parasitic infections, imatinib for myeloproliferative forms, or combination immunosuppressive therapy for eosinophilic granulomatosis with polyangiitis or hypereosinophilic syndromes.^107^ Fulminant forms, including giant cell myocarditis, are typically treated with high-dose pulse corticosteroids, cyclosporine, and T-cell depleting therapies, such as antithymocyte globulin.^108^

Emerging data from clinical trials have begun to assess the efficacy of targeted anti-inflammatory therapies in myocarditis. The ARAMIS trial demonstrated the safety of anakinra in patients with acute myocarditis, but did not show a reduction in short-term complications over 28 days.^95^ The ongoing MYTHS trial (NCT05150704) is evaluating the efficacy of pulsed intravenous methylprednisolone compared with standard therapy in a larger cohort of patients with acute myocarditis.

Inflammatory cardiomyopathy, defined as myocarditis associated with ventricular dysfunction and adverse remodelling, remains a major clinical challenge, particularly when complicated by heart failure or arrhythmias.^109^ Despite recent progress in elucidating disease mechanisms, significant knowledge gaps persist regarding viral persistence, immune dysregulation, and genetic susceptibility. Novel research strategies are being developed to address these limitations, including phenomapping (integration of clinical, imaging, and histological features), next-generation sequencing, and epigenetic profiling. Experimental therapies under investigation include IL-1β and IL-17 pathway inhibitors, as well as cell-based immunomodulation.^110^ In fulminant cases, mechanical circulatory support not only stabilizes hemodynamics but may also modulate inflammation and limit irreversible myocardial injury, serving as a bridge to recovery or transplantation.^111^

In summary, in myocarditis, treatment remains empirical, with growing interest in etiology-specific and targeted approaches. Preliminary results from recent trials of such approaches have confirmed feasibility and safety, but robust data supporting efficacy are still lacking, underscoring the need for large, well-designed, mechanistically informed studies.

## Inflammation, atrial fibrillation and stroke

Inflammation plays a key role in the pathogenesis and maintenance of atrial fibrillation (AF) (*Table 5*). Inflammatory signalling contributes to structural and electrical remodelling of the atrial myocardium, promoting fibrosis, conduction abnormalities, and increased arrhythmogenic potential.^115^ This mechanistic link has prompted interest in targeting inflammatory pathways to reduce AF recurrence and associated ischaemic complications.

**Table 5:** Key randomized controlled trials of anti-inflammatory therapies in patients with atrial fibrillation

Trial	Study design	Population	Intervention	Active comparator	Primary endpoint(s)	Result
IMPROVE-PVI,^112^ 2024	Randomized, double-blind, placebo-controlled	199 undergoing ablation for AF	Colchicine 1.2 mg/day for 10 days	Placebo	AF recurrence (2 weeks and 3 months)	HR, 0.98; (0.59–1.61; *P* = 0.92)
CONVINCE,^113^ 2024	Randomized, open-label, blinded-endpoint	3154 with NC stroke/TIA	Colchicine 0.5 mg/day added to standard care	Standard care alone	Stroke/TIA	HR, 0.84; (0.68–1.05)
CHANCE-3,^114^ 2024	Randomized, double-blind, placebo-controlled	8343 high-risk stroke/TIA	Colchicine: 1 mg/day for 1–3 days, then 0.5 mg/day for 90 days	Placebo	Stroke recurrence at 90 days	HR, 0.98; (0.83–1.16; *P* = 0.79)
CASPER, ACTRN12621001408875	Randomized, double-blinded, placebo-controlled	1500 high-risk stroke with persistent inflammation (elevated hs-CRP)	Colchicine 0.5 mg/day	Standard care	Major adverse cardiovascular event	—

AF, atrial fibrillation; CI, confidence interval; HR, hazard ratio; hs-CRP, high-sensitivity C-reactive protein; NC, non-cardioembolic; TIA, transient ischaemic attack.

### Atrial fibrillation and post-operative risk

Colchicine has been evaluated in several randomized trials for its potential to improve outcomes in AF management. In the IMPROVE-PVI trial, 199 patients undergoing catheter ablation were randomized to colchicine or placebo in addition to standard therapy.^112^ While colchicine did not significantly reduce AF recurrence at 2 weeks or 3 months, it led to a reduction in post-ablation pericarditic chest pain, albeit with increased gastrointestinal adverse events. Similar findings emerged from the COP-AF and COCS trials, where colchicine modestly reduced the incidence of post-operative AF following cardiac surgery, but the magnitude of benefit has not been sufficient to alter guideline recommendations.

In the structural heart intervention setting, the abovementioned Co-STAR trial (NCT04870424) investigated colchicine following transcatheter aortic valve implantation, hypothesizing a benefit on the composite of new-onset AF and need for permanent pacemaker implantation. The composite endpoint was reduced in the colchicine group, but neither component reached statistical significance individually. The trial was terminated early due to a numerically higher stroke rate in the colchicine arm, which investigators attributed to baseline differences in cerebrovascular disease rather than a direct drug effect.

In summary, current evidence does not support the routine use of colchicine to prevent AF recurrence. Some benefit may exist in specific subgroups, such as patients with post-operative AF or elevated inflammatory markers, but trial results are not conclusive.

### Non-cardioembolic stroke

Beyond AF, colchicine has been explored for secondary prevention in patients with ischaemic stroke. In the CONVINCE trial, colchicine reduced CRP levels in patients with non-cardioembolic stroke or transient ischaemic attack (TIA), but the primary composite outcome (recurrent ischaemic stroke, MI, cardiac arrest, or hospitalization) was not significantly reduced, partly due to premature trial termination related to funding constraints during the COVID-19 pandemic.^113^ Similarly, the CHANCE-3 trial, enrolling patients with minor-to-moderate ischaemic stroke or TIA and elevated high-sensitivity CRP, found no significant reduction in event recurrence at 90 days with colchicine treatment.^114^

A meta-analysis of six randomized trials (*n* = 14 934), including patients with prior stroke or CAD, found that low-dose colchicine reduced the risk of ischaemic stroke by 27% compared with placebo or standard care.^116^ The benefit was consistent across subgroups defined by age, sex, diabetes status, and statin use. Notably, 3144 patients had a history of ischaemic stroke or high-risk TIA, and 5522 had stable CAD. No increase in serious adverse events was observed. Because this study did not focus specifically on patients with stroke, it cannot provide robust evidence supporting the use of colchicine for this population.

## Gaps in knowledge and emerging strategies

Inflammation is a recognized contributor to the pathogenesis of various manifestations of CVD, including atherosclerosis, heart failure, valvular heart disease, coronary microvascular dysfunction, pericarditis, myocarditis, AF, and stroke.^21^ Consequently, several anti-inflammatory strategies are under investigation across multiple cardiovascular domains. Key considerations in implementing such therapies include potential class effects, optimal dosing and timing, and the development of novel agents or combination regimens. The recognition of inflammation as a component of residual cardiovascular risk supports the rationale for personalized or multi-targeted therapeutic approaches. Whether the observed benefits of anti-inflammatory therapies derive from shared mechanisms or unique pharmacodynamic properties remains unresolved.^117^

### Colchicine in the post-myocardial infarction: open questions and knowledge gaps

Colchicine is currently the only anti-inflammatory drug recommended for secondary cardiovascular prevention in CAD.^24,49,118^ However, its role may be re-evaluated in future guidelines following the publication of the CLEAR trial, which did not demonstrate a significant benefit of colchicine after MI.^39^ Several trial-specific factors merit consideration when putting the CLEAR trial into perspective. First, early colchicine administration too early after an STEMI may impair reparative inflammation and endothelial healing following percutaneous coronary intervention. Second, in CLEAR, a trend towards benefit was evident before the onset of the COVID-19 pandemic, suggesting potential interference from biological or operational disruptions. Third, patients receiving higher colchicine doses appeared to derive more benefit, suggesting that the 0.5 mg/day regimen may be suboptimal in the context of ACS.

The optimal colchicine dose and timing remain uncertain. Most trials in stable CAD employed low-dose regimens.^117^ However, given colchicine's dose-dependent effects in other settings (e.g. pericarditis), the potential benefit of higher doses in acute MI, characterized by high inflammatory burden, warrants further study. Experimental evidence suggests that inflammation peaks within hours of MI onset and contributes to myocardial injury.^119^ Anti-inflammatory interventions appear most effective when administered between 3- and 12-h post-symptom onset. As noted above, very early administration (e.g. immediately post-revascularization) may disrupt reparative processes, whereas delayed treatment may miss the inflammatory peak.^119^ Nonetheless, a meta-analysis of 28 trials (*n* = 44 406) found no significant interaction between timing and MACE, suggesting that timing may not be an independent determinant of efficacy.^117^

### Evidence on colchicine from recent meta-analyses

A recent meta-analysis by d’Entremont et al., including nine randomized trials (*n* = 30 659) reported a significant reduction in MACE with colchicine vs. control (HR 0.88; 95% CI 0.81–0.95; *P* = 0.002).^120^ This was primarily driven by reductions in MI (HR 0.84; 95% CI 0.73–0.97; *P* = 0.016) and coronary revascularization (HR 0.83; 95% CI 0.74–0.94; *P* = 0.002). No significant effect was observed for cardiovascular death (HR 0.94; 95% CI 0.78–1.13; *P* = 0.51) or stroke (HR 0.90; 95% CI 0.80–1.02; *P* = 0.09). The number needed to treat was 149 for MI prevention and 100 for revascularization, whereas the number needed to harm for gastrointestinal hospitalization was 182 (RR 1.35; 95% CI 1.10–1.66; *P* = 0.004). These numbers suggest that the use of colchicine, at best, should not be considered routine but selective.

**Figure 2.**
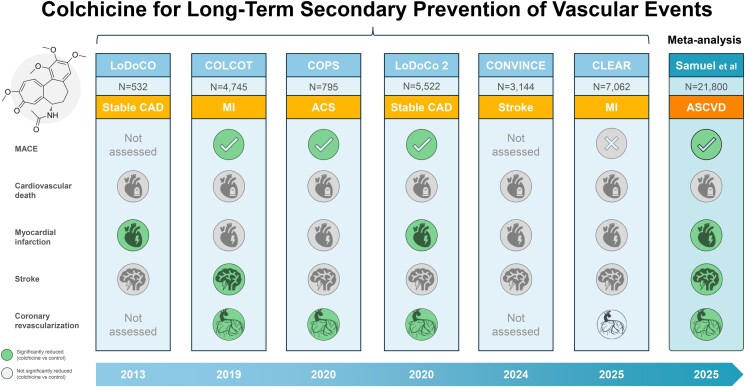
Trials of colchicine for long-term secondary prevention of vascular events. In a recent meta-analysis of six randomized controlled trials of secondary prevention in vascular disease patients (*N* = 21 800) from Samuel et al., the administration of colchicine resulted in a reduction of major cardiovascular events, fewer myocardial infarctions, ischaemic strokes, and recurrent coronary revascularizations. ACS, acute coronary syndromes; CAD, coronary artery disease; MACE, major cardiovascular events; MI, myocardial infarction.

In a separate meta-analysis by Samuel et al., which excluded the CHANCE-3 trial due to short follow-up and used a different endpoint definition, a more pronounced MACE reduction was observed (HR 0.75; 95% CI 0.56–0.93; *P* < 0.001) (*Figure 2*).^121^ This analysis reported greater effect sizes for MI (HR 0.71; 95% CI 0.51–0.91) and ischaemic stroke (HR 0.63; 95% CI 0.34–0.92). However, reliance on a random-effects model and inclusion of smaller trials may have led to overestimation. Heterogeneity in trial populations further limits the generalizability of these results.

### Emerging anti-inflammatory strategies in cardiovascular disease

Promising alternative anti-inflammatory targets are under investigation. IL-6 inhibition, particularly with ziltivekimab, may offer broader immunomodulatory effects than colchicine or IL-1 blockade. TNF-α inhibition remains controversial due to limited efficacy and concerns about immunosuppression. Targeting the NLRP3 inflammasome is another promising but experimental approach. Immune-modulatory strategies, such as regulatory T-cell expansion or cardiovascular-adapted checkpoint inhibitors, aim to restore immune balance without compromising host defenses.^122,123^ RNA-based therapies, including small interfering RNA and antisense oligonucleotides, may enable precise targeting of inflammatory pathways at the transcriptional level.^124^

Finally, several trials test strategic combinations or factorial designs rather than single-drug interventions. The CLEAR SYNERGY trial investigated colchicine and spironolactone for their respective effects on acute inflammation and post-MI remodelling, while the MACT trial explored combined colchicine and P2Y_12_ inhibition after MI.^39,125^ These studies are interesting as they aimed to exploit off-target effects to enhance cardiovascular benefit through synergistic mechanisms.

## Conclusions

Inflammation is now recognized as a central pathogenic mechanism in CVD, contributing to conditions such as atherosclerosis, heart failure, and aortic stenosis. Decades of mechanistic and clinical research have established inflammation as a modifiable driver of disease progression rather than a secondary phenomenon. This paradigm shift has led to the development of targeted anti-inflammatory therapies, advancing beyond broad-spectrum agents such as colchicine towards more selective, pathway-specific interventions.

Ongoing trials are assessing inflammation-targeted strategies not only in ACS and stable CAD but also in areas with unmet therapeutic needs, including microvascular dysfunction, HFpEF, and moderate aortic stenosis. In these contexts, early modulation of inflammatory pathways may prevent structural remodelling and disease progression. The relevance of these strategies is heightened by the rising prevalence of such conditions in aging populations.

Among emerging therapies, monoclonal antibodies targeting ILs, particularly IL-6, represent a promising approach. Their use in high-risk individuals with persistent systemic inflammation and frequent hospitalizations may reduce recurrent events and healthcare utilization. The clinical impact of these therapies will depend on precise patient selection, appropriate timing of intervention, and dose optimization.

## Data Availability

The data underlying this review were taken from the quoted references.
